# CDK5 Knockdown inhibits proliferation and induces apoptosis and Cell Cycle Arrest in Human Glioblastoma

**DOI:** 10.7150/jca.53981

**Published:** 2021-05-10

**Authors:** Yan Zhou, Xuan Wang, Peng Lv, Hao Yu, Xiaobing Jiang

**Affiliations:** 1 Department of Neurosurgery, Union Hospital, Tongji Medical College, Huazhong University of Science and Technology, Wuhan 430022, China.; 2 Department of Neurosurgery, Suizhou Hospital, Hubei University of Medicine, Suizhou, Hubei, 441300, China.

**Keywords:** bioinformatics analysis, CDK5, gene set enrichment analysis, glioma, metabolism

## Abstract

**Aims:** Gliomas are the most common malignant brain neoplasms with high recurrence and lethality rates. Recently, studies have reported that cyclin-dependent kinase 5 (CDK5) is involved in tumorigenesis. Herein, we applied bioinformatics analysis to determine the clinical value of CDK5 in patients with glioma and examined the effects of CDK5 on glioblastoma cell proliferation, apoptosis, and cell cycle *in vitro*.

**Methods:** Gene expression profiles containing clinical data of low-grade glioma (LGG) and glioblastoma cohorts were obtained from The Cancer Genome Atlas database and analyzed to determine the association between CDK5 expression and glioma clinicopathological characteristics. Kaplan-Meier survival analysis was performed for prognosis analysis. Gene set enrichment analysis (GSEA) was used to identify the biological pathways involved in differential CDK5 expression. *In vitro* experiments were performed to explore the effects of CDK5 on glioma cell functions.

**Results:** CDK5 expression was substantially higher in glioblastoma than in LGG. GSEA showed that some metabolism-related pathways were associated with the high CDK5 expression phenotype. *In vitro* experiments showed that CDK5 knockdown impaired cell proliferation and colony formation ability, and induced apoptosis and cell cycle arrest.

**Conclusion:** CDK5 may act as a potential biomarker of glioma progression and a valid target for glioma therapy.

## Introduction

Gliomas are the most common malignant brain neoplasms 1. According to the World Health Organization grading system, gliomas can be classified into four grades, and glioblastoma (IV) is the most aggressive 2. Although advances in glioma treatment have been achieved in the past decades, the effects of treatment, especially on glioblastomas, remain unsatisfactory 3. The median survival time of patients with glioblastoma remains less than 15 months after diagnosis, indicating that it is crucial to explore the mechanism of glioblastoma progression and establish treatments that are more effective 4.

Cyclin-dependent kinase 5 (CDK5), a proline-directed serine/threonine kinase and a member of the CDK family, is abundantly present in the central nervous system 5. In the past decade, CDK5 has been shown to play an important role in neurons. CDK5 contributes to neurite outgrowth, neuronal migration, and neuronal cell survival 6, 7, 8.

Recent studies have reported that CDK5 is dysregulated in various tumor cells, and it participates in tumorigenesis 9. Zeng et al. 10 reported that high CDK5 expression promoted lung cancer cell proliferation and metastasis. Zhou et al. 11 showed that CDK5 generated vasculogenic mimicry in a lung cancer cell line via the FAK/AKT signaling pathway. Zhuang et al. 12 reported that CDK5 could directly phosphorylate ERK5 and promote progression of colorectal cancer carcinogenesis by CDK5-ERK5-AP-1 axis. Yushan et al. 13 demonstrated that CDK5 was positively correlated with the advanced tumor grade of glioma based on 152 clinical specimens. However, previous studies on the role of CDK5 in glioma are still limited, and the clinical prognostic value of CDK5 remains unclear.

In this study, we explored the prognostic value of CDK5 expression in patients with glioma based on The Cancer Genome Atlas (TCGA) and Gene Expression Omnibus (GEO) databases. Experiments were conducted to validate bioinformatics data reliability and the connection between CDK5 expression and clinicopathological features of gliomas.

## Methods

### Public database and bioinformatics analysis

High-throughput sequencing data (HTSeq-FPKM) were directly downloaded from TCGA (http://cancergenome.nih.gov) 14. Through the Genomic Data Commons (GDC) interface, we downloaded the detailed clinical information of all glioma samples including low-grade glioma (LGG, II-III) and glioblastoma cohorts. The microarray data (as validation) were downloaded from the GEO public database (https://www.ncbi.nlm.nih. gov/geo) 15 under accession number GSE16011 16, which contained a large sample size and patients had not received any treatment before the microarray examination. Patients with complete information (CDK5 mRNA expression and clinicopathological features) were enrolled into the corresponding analysis.

### Gene set enrichment analysis (GSEA)

Based on TCGA, GSEA 17 was conducted to identify the mechanisms underlying the interaction between CDK5 expression and glioma progression, and determine whether some biological pathways in high and low CDK5 expression cohorts exhibited statistically significant differences. For each analysis, gene set permutations were enforced 1000 times. Gene sets with a false discovery rate (FDR) <0.05 and a normal P-value <0.05 were considered significantly enriched.

### Cell lines and cell culture reagents

Normal human astrocytes (NHAs) and U87MG, U251MG, and U138MG human glioblastoma cell lines were obtained from our laboratory. NHAs were cultured in astrocyte medium. U251MG and U87MG were cultured in Dulbecco's Modified Eagle Medium (DMEM; GIBCO, Invitrogen, CA, USA) supplemented with 2 mM L-glutamine, penicillin (100 U/mL), and 10% fetal bovine serum (FBS; GIBCO, Invitrogen).

### Transfection

A lentivirus expressing short hairpin RNA (shRNA) that targeted CDK5 was designed and synthesized by Shanghai GeneChem Co., Ltd. (Shanghai, China). U251MG cells (5×10^4^ cells per well) were grown in 6-well plates (Corning, NY, USA) 24 h before infection. Lentiviruses containing CDK5 shRNA (sh-CDK5) and scrambled shRNA (sh-NC) were individually transfected into U251MG cells, according to the manufacturer's instructions. The infection efficiency was determined by the intensity of green fluorescence protein after 72 h. The knockdown efficiency was examined using quantitative reverse transcription-polymerase chain reaction (qRT-PCR) and western blotting 96 h after transfection.

### RNA extraction and qRT-PCR

Total RNA was extracted using TRIzol reagent, and qRT-PCR was performed for samples with 260/280 values between 1.8 and 2.0 using a qRT-PCR cDNA kit. Real-time PCR reactions were performed on an ABI 7500 instrument. Primers synthesized by Wuhan Tsingke Biotech Co., Ltd. (China) were as follows: CDK5 up: GGAAGGCACCTACGGAACTG and CDK5 down: GGCACACCCTCATCATCGT and glyceraldehyde-5-phosphate dehydrogenase (GAPDH) up: AGCCACATCGCTCAGACAC and GAPDH down: CTCGCTCCTGGAAGATGGT. The PCR reaction conditions were: 95 °C for 10 min, followed by 40 cycles at 95 °C for 15 s and 59 °C for 40 s. Relative quantitative analysis was performed using the 2^-ΔΔCt^ method, where Ct is threshold cycle. The experiment was repeated thrice.

### Western blotting

U251MG cells were homogenized in RIPA lysis buffer, which contained a protease inhibitor mixture (Beyotime, Shanghai, China), on ice for 30 min, removed from the 6-well plates using a cell scraper, and centrifuged at 12,000 rpm in a 4 °C Eppendorf microfuge (Germany) for 10 min. Loading buffer was added to the protein lysate and boiled for 10 min. Equal amounts of protein were loaded, separated via 10% SDS-PAGE, and transferred to a polyvinylidene fluoride membrane (Millipore, Bedford, MA, USA) via electroblotting for 90 min. The membranes were blocked with 5% skim milk for 1 h and then incubated overnight with primary antibodies to CDK5 (Abcam, Cambridge, MA, USA) and β-Actin (Proteintech, Wuhan, China) at 4 °C along with appropriate secondary antibodies. The enhanced chemiluminescence system (Beyotime, Shanghai, China) was used to identify the reactive bands, according to the manufacturer's instructions.

### Colony formation assay

Transfected and control cells (n = 500) were seeded in 6-well plates and cultured in DMEM (high glucose) (Thermo Fisher Scientific, Shanghai, China) supplemented with 10% FBS (GIBCO) and 1% antibiotics (penicillin-streptomycin, GIBCO) for 10 d. The colonies were fixed with 75% ethanol, stained with 0.1% crystal violet solution, imaged, and counted.

### Annexin V-APC Apoptosis assay

An Annexin V-APC Apoptosis Detection Kit (eBioscience, CA, USA) was used for cell apoptosis analysis. First, U251MG cells were infected with lentiviruses expressing sh-CDK5 or sh-NC. After 4 d, cells were harvested, washed with phosphate-buffered saline (PBS), and resuspended in staining buffer to obtain a final density of 1×10^7^/mL. Next, Annexin V-APC was added to the cell suspensions (100 µL) and incubated at room temperature for 10-15 min. Signals were detected with a flow cytometer (Millipore Guava easyCyte HT).

### Cell cycle analysis

U251MG cells infected with lentivirus expressing sh-CDK5 or sh-NC were cultivated in 6-cm dishes. When the density reached 80% coverage, cells were collected and fixed with pre-cooled 70% alcohol for 1 h. Cells were then washed with PBS and stained with a buffer containing 40×propidium iodide (PI) stock (2 mg/mL), 100×RNase stock (10 mg/mL), and 1×PBS at a dilution of 25:10:1,000. Flow cytometry was performed using the Millipore Guava easyCyte HT flow cytometer for cell cycle analysis.

### EdU incorporation assay

U251MG cells (2×10^3^ cells per well) transfected with sh-CDK5 or sh-NC were cultured in 96-well plates at 37 °C for 72 h and then exposed to 5-ethynyl-20-deoxyuridine (EdU, 50 μmol/L, RiboBio Co., China) for an additional 2 h. The cells were fixed with 4% formaldehyde for 30 min and treated with 0.5% Triton X-100 at room temperature for 20 min. After washing with PBS thrice, 1×Apollo® reaction cocktail (100 µL) was added to each well and incubated for 30 min. Subsequently, the DNA contents of the cells in each well were incubated with 5 µg/mL Hoechst 33342 (100 µL) for 30 min, washed with PBS thrice, and imaged under a fluorescent microscope.

### Statistical analysis

The statistical analyses of TCGA and GSE16011 data were performed using R software v3.5.1. and GraphPad Prism 7 software. To analyze the potential relationship between CDK5 expression and the clinicopathological features of gliomas, Mann-Whitney U and logistic regression tests were performed. The Kaplan-Meier method was used to evaluate the effects of CDK5 expression on the overall survival (OS) of glioma patients. All experiments were performed at least thrice. Data are presented as the mean ± standard deviation. Student's t-test was used to determine statistical differences between two groups. One-way analysis of variance (ANOVA) was used to compare three groups. P <0.05 was used to determine the significance level.

## Results

### The CDK5 transcription level in gliomas based on TCGA

**Figure 1:**
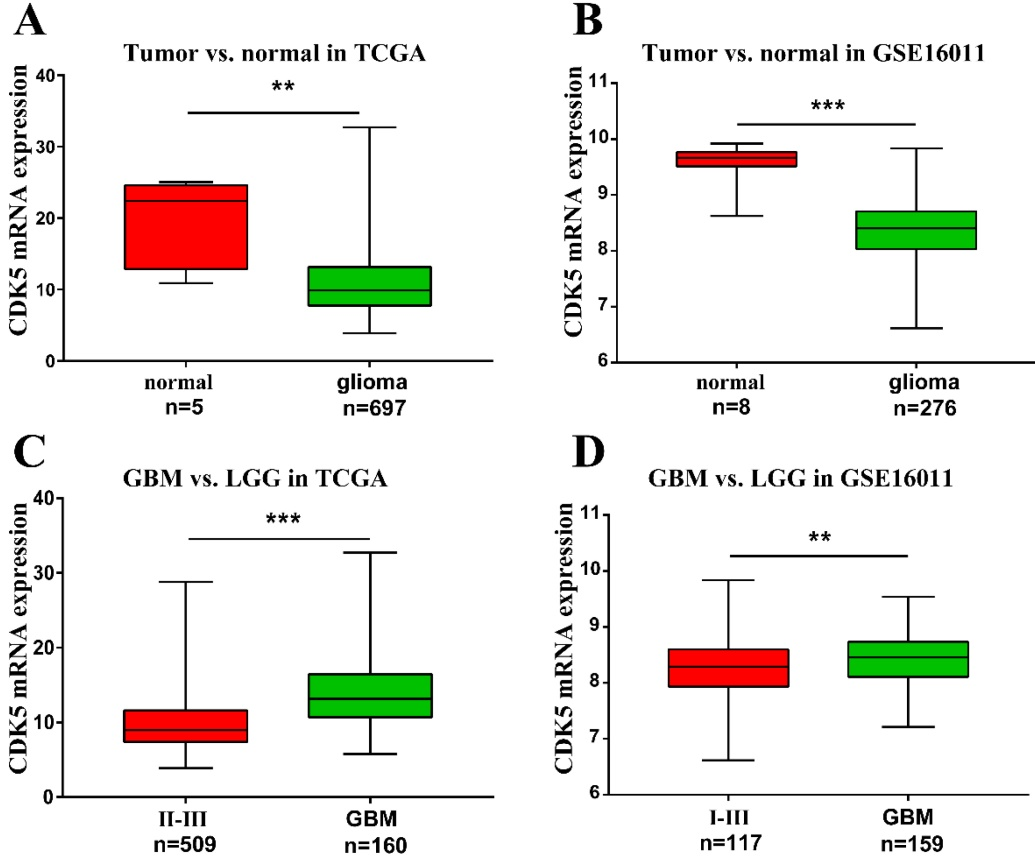
** Transcription level of CDK5 in gliomas based on TCGA and GSE16011.** (A, B) CDK5 expression levels in glioma tissues, compared with in normal tissues, are shown. (C, D) CDK5 expression levels in glioblastomas (IV), compared with in low-grade gliomas (II-III or I-III), are shown.

To investigate the role of CDK5 in gliomas, the gene expression profiles and clinical data of patients with glioma were obtained from TCGA and GSE16011 datasets. A total of 1114 and 276 glioma cases with clinical data were available from TCGA and GSE16011 dataset respectively. The number of patients with CDK5 transcript data is 697 (5 normal tissues). All clinical characteristics were summarized in Table S1. By comparing tumor and normal tissues, we found that CDK5 mRNA levels were lower in glioma tissues than in normal tissues (Figure 1A, B). This might be due to the abundance of CDK5 in neurons 6. However, CDK5 expression was significantly higher in glioblastoma than in LGG (II-III or I-III) (Figure 1C, D), indicating that CDK5 played an important role in disease progression.

### Association between CDK5 expression and the clinicopathological features of gliomas

**Figure 2:**
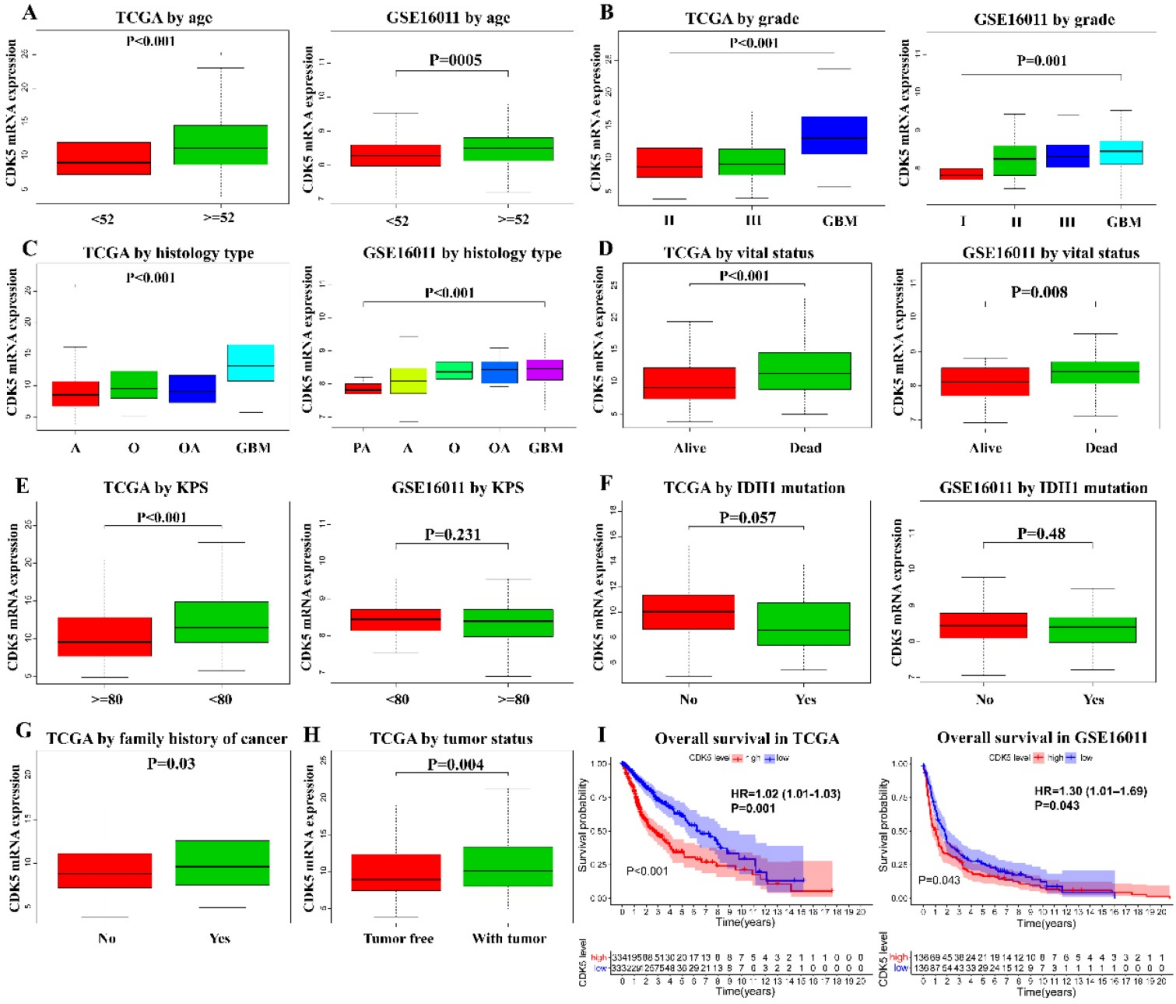
** Association between CDK5 expression and the clinicopathological features of patients with glioma in TCGA and GSE16011 cohorts.** The clinical features include (A) age, (B) tumor grade, (C) histological type, (D) vital status, (E) KPS, (F) IDH1 mutation, (G) family history of cancer, and (H) tumor status. (I) The effects of CDK5 expression on the overall survival of patients are shown.

To explore the CDK5 expression pattern in gliomas, data on the clinical features of patients with glioma obtained from TCGA and GSE16011 were analyzed. As shown in Figure 2A-D, CDK5 expression level was significantly associated with age, tumor grade, histological type and vital status in both TCGA and GSE16011 cohorts. Additionally, CDK5 expression was also significantly correlated with Karnofsky Performance Status (KPS) (P <0.001), family history of cancer (P = 0.03), and tumor status (P = 0.004) in TCGA cohorts, while no significant differences were observed for KPS (P = 0.231) and IDH1 mutation (P = 0.48) in GSE16011 cohorts (Figure 2E-H).

Logistic regression analysis showed that CDK5 expression was associated with poor prognostic clinicopathological characteristics (Table 1). When studying the relationship between age and CDK5 expression in TCGA, the samples enrolled into logistic regression analysis should contain age data and RNA-seq data. The number of such samples is 670, while the number of enrolled samples might be different in the analyses for other clinical features. In TCGA, CDK5 expression level in glioma was significantly associated with age {≥52 vs. <52, odds ratio (OR) = 2.47, 95% confidence interval (CI) [1.80-3.41], P <0.001}, vital status (dead vs. alive, OR = 2.36, 95% CI [1.69-3.29], P <0.001), grade (IV vs. II, OR = 6.67, 95% CI [4.29-10.6], P <0.001), histological type (glioblastoma vs. astrocytoma, OR = 9.87, 95% CI [6.09-16.4], P <0.001; glioblastoma vs. oligoastrocytoma, OR = 4.65, 95% CI [2.83-7.74], P <0.001; glioblastoma vs. oligodendroglioma, OR = 5.85, 95% CI [3.71-9.38], P <0.001), tumor status (with tumor vs. tumor‐free, OR = 1.72, 95% CI [1.21-2.44], P = 0.002), KPS (<80 vs. ≥80, OR = 2.82, 95% CI [1.65-4.99], P <0.001), ethnicity (Hispanic or Latino vs. not Hispanic or Latino, OR = 0.342, 95% CI [0.149-0.720], P = 0.007), and IDH1 mutation (yes vs. no, OR = 0.428, 95% CI [0.185-0.954], P = 0.0414). No significant differences in the sex, family history of cancer, and grade (III vs. II) subgroups were observed. Similar results were also obtained from GSE16011 dataset, indicating that CDK5 may be liable for glioma progression from a low-grade malignancy to an advanced malignancy.

### Survival outcomes

To investigate the predictive value of CDK5 in glioma prognosis, we analyzed CDK5 expression and the OS of glioma patients. For patients with complete OS data, Kaplan-Meier analysis was performed. As shown in Figure 2I, patients with glioma with higher CDK5 expression had a worse prognosis in TCGA cohorts {hazards ratio (HR) 1.02, 95% CI [1.01-1.03], P = 0.001}, which was validated in GSE16011 cohorts {hazards ratio (HR) 1.30, 95% CI [1.01-1.69], P = 0.043}.

### CDK5-related signaling pathways were identified based on GSEA

**Figure 3:**
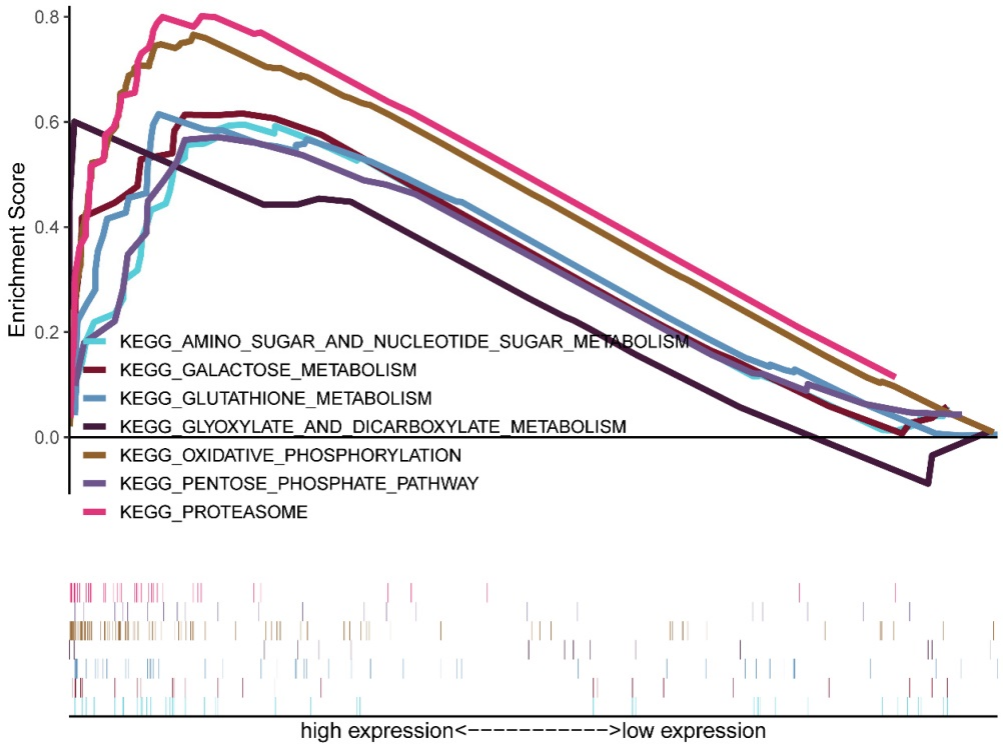
Enrichment plots obtained based on gene set enrichment analysis.

GSEA was conducted between the low and high CDK5 expression phenotype groups to identify the signaling pathways involved in glioma progression based on TCGA. Significant differences (FDR <0.05) were observed in the enrichment analysis. Several signaling pathways, especially metabolism-related pathways, including amino sugar and nucleotide sugar metabolism, galactose metabolism, proteasome, glyoxylate, and dicarboxylate metabolism, oxidative phosphorylation, pentose phosphate pathway, and glutathione metabolism, were enriched in the high CDK5 expression cohort (Figure 3, Table 2).

### CDK5 exerted promotional effects on glioma cell proliferation *in vitro*

**Figure 4:**
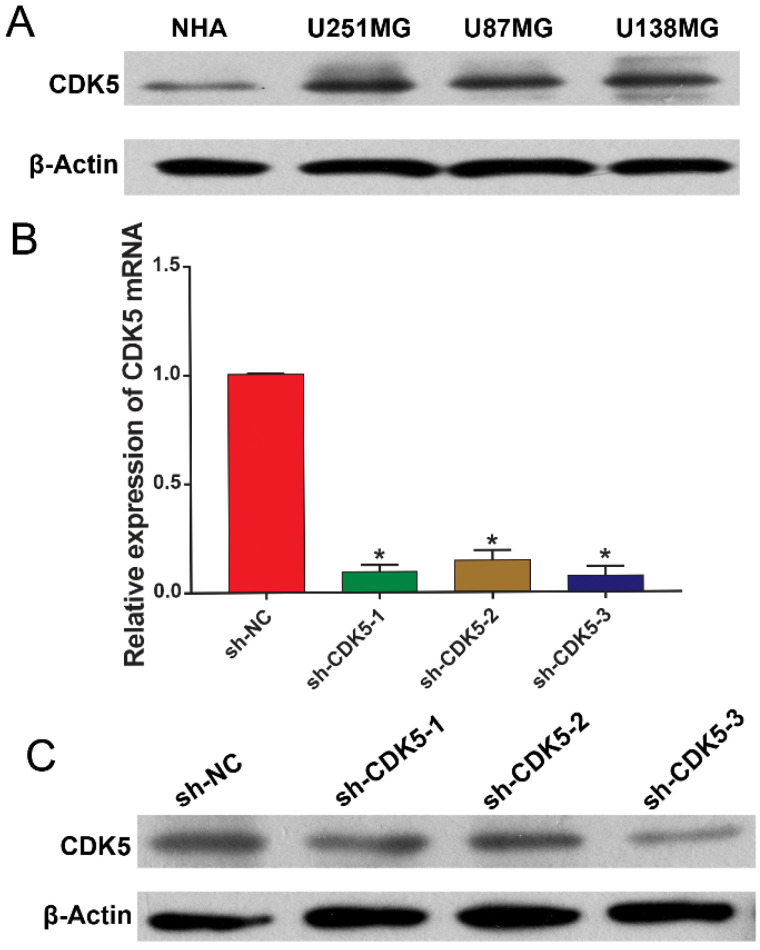
(A) CDK5 expression levels in glioma cell lines (U251MG, U87MG, and U138MG) and normal human astrocytes are shown. (B and C) CDK5 mRNA and protein levels in CDK5-silenced U251MG cells are shown. *P <0.05.

Next, we sought to elucidate the biological functions of CDK5 in glioma cells *in vitro*. CDK5 in glioma cell lines (U251MG, U87MG, and U138MG) and NHAs was subjected to immunoblotting. As shown in Figure 4A, the CDK5 expression levels were significantly higher in the glioma cell lines than in the NHAs; therefore, U251MG cell line was selected for further analysis. CDK5 mRNA and protein expression in U251MG cells transfected with three sets of sh-CDK5 (Figure 4B, C) was examined. We selected sh-CDK5-3, which showed the most significant decline, for follow-up experiments.

**Figure 5:**
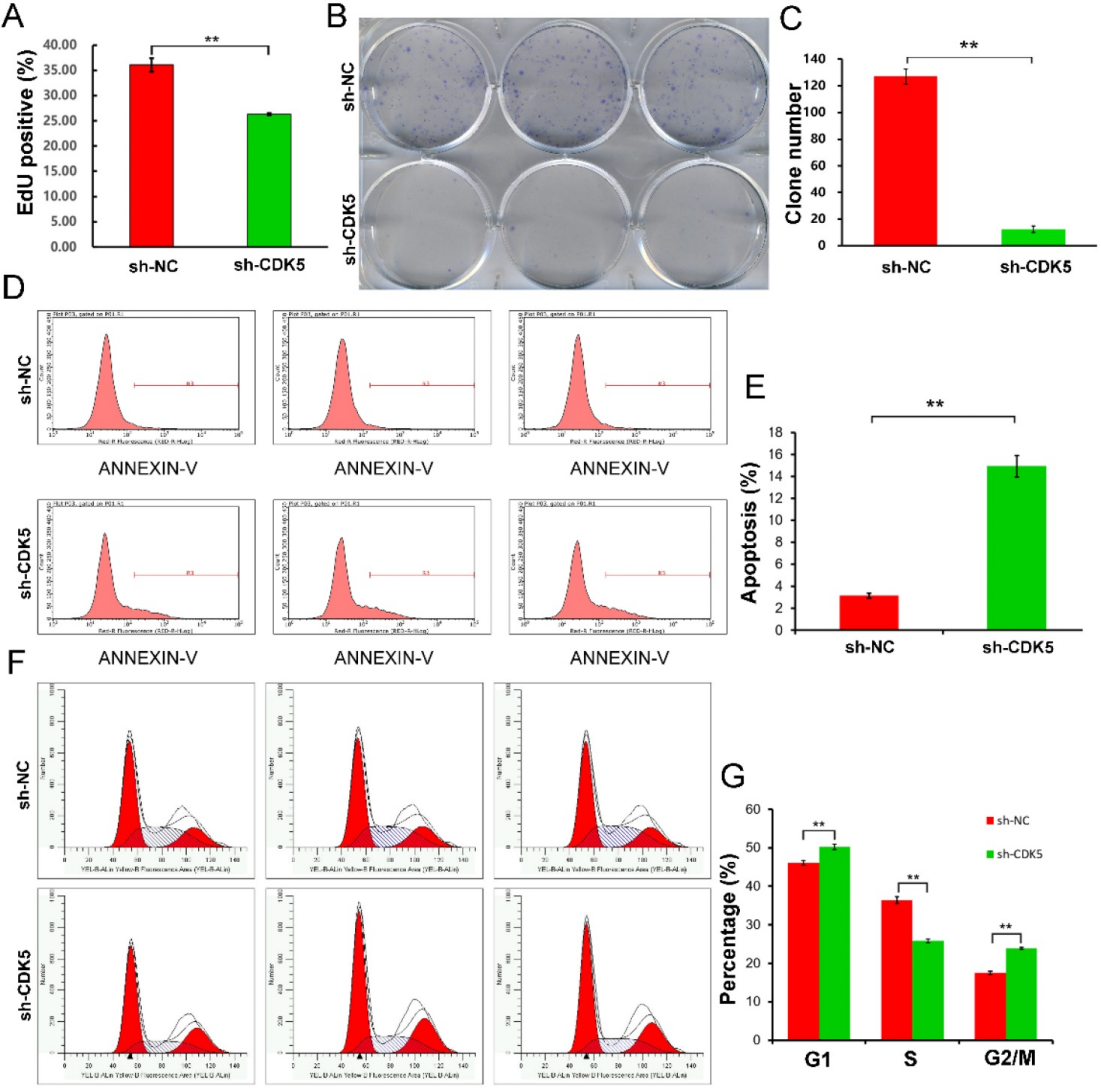
** Cell proliferation, apoptosis, and cell cycle assays.** Proliferation was quantified using EdU and colony formation assays. (A) EdU staining showed that CDK5 knockdown, compared with control treatment, decreased the proportion of EdU-positive cells. Data are presented as mean ± standard deviation (SD); **P <0.01 vs. control, unpaired t-test. (B and C) Colony formation assay showed that the clonogenic ability of U251MG cells was impaired due to CDK5 knockdown. Quantitative results represent mean ± SD of three separate experiments; **P <0.01, unpaired t-test. (D and E) Flow cytometry analysis of apoptotic rates of U251MG cells treated with scrambled (sh-NC) or CDK5 (sh-CDK5) short hairpin RNA was performed. Quantitative data are presented as mean ± SD; **P <0.01, unpaired t-test. (F and G) Cell cycle distribution in U251MG cells infected with lentivirus expressing sh-NC or sh-CDK5 was analyzed by flow cytometry. Quantitative graph showed the mean ± SD values of the proportions of cells in the G1, S, and G2/M phases from three separate experiments; **P <0.01, unpaired t-test.

In this study, cell proliferation was evaluated using the EdU and colony forming assays. EdU assay showed that sh-CDK5 cells, compared to sh-NC cells, inhibited cell proliferation significantly (P <0.01) (Figure 5A). Colony formation assay displayed a similar result; CDK5 knockdown significantly inhibited colony formation (P <0.01) (Figure 5B, C).

### U251MG cell apoptosis was promoted by CDK5 knockdown

Tumor cells show strong resistance to cell death. In this study, Annexin V-APC assay and flow cytometry were performed to evaluate apoptosis of U251MG cells infected with lentivirus expressing sh-CDK5. Apoptosis was detected in 3.16% of cells treated with sh-NC and 14.93% of cells treated with sh-CDK5 (Figure 5D, E).

### Cell cycle arrest was induced by CDK5 knockdown

Subsequently, flow cytometry and PI staining were performed to determine the alteration in the cell cycle (Figure 5F, G). It was shown that the proportion of U251MG cells in the G1 and G2/M phases was significantly higher and that in the S phase was significantly lower in the sh-CDK5 group than in the sh-NC group, indicating that CDK5 knockdown inhibited tumor proliferation.

## Discussion

Malignant evolution, a hallmark of tumors, is essential for cancer progression. Without timely and effective therapy, a glioma may progress from low-grade to high-grade, exacerbating the prognosis 18, 19. Some, but not all, molecules involved in glioma progression have been identified. In this study, analyses results from TCGA and GSE16011 datasets showed that high CDK5 expression level was correlated with poor prognosis and advanced clinicopathological parameters such as age, tumor grade and histological type. GSEA showed that some metabolism-related pathways were differentially enriched in the high CDK5 expression phenotype group. Additionally, *in vitro* experiments were performed. EdU incorporation and colony forming assays showed that CDK5 promoted glioma cell proliferation and colony formation capacity. Moreover, CDK5 knockdown promoted cell apoptosis and altered the cell cycle distribution. These results indicated that targeting CDK5 could be a therapeutic strategy to inhibit glioma progression.

Although the role of CDK5 in gliomas has been previously investigated, relevant studies remain limited. CDK5, as a neuron-specific kinase, has been known to be abundantly present in the brain 6, 20. Yushan et al. 13 demonstrated that CDK5 was positively correlated with the pathological grade and Ki-67 of glioma based on 152 clinical specimens. However, the prognostic value of CDK5 wasn't explored in their study due to the lack of follow-up time data. In our study, we had a large sample size obtained from two public databases with full clinical information and we found that patients with CDK5-high had shorter survival time. Apart from that, Yushan's study also demonstrated that the positive CDK5 ratio was higher in glioma tissues than in normal brain which was contrary to the results of our study and Liu's research 21. Therefore, additional studies with fewer confounding factors must be conducted to confirm the expression level of CDK5 in both tumor and normal tissues. Dorand et al. 22 reported that CDK5 increased PD-L1 expression in tumors and allowed medulloblastomas to evade immune elimination, indicating that CDK5 might be a target for tumor immunotherapy. In this study, we found that CDK5 was related to several tumor metabolism-related pathways based on GSEA, which provided a new direction that CDK5 may be a target for tumor metabolism and contribute to the development of glioma treatments 23, 24. Mukherjee et al. 25 revealed that CDK5 promoted glioma stem cell self-renewal and Liu et al. 21 reported that phosphorylation of PIKE-A by CDK5 facilitated glioblastoma cell invasion and migration. In our study, we assessed the influence of CDK5 on cell proliferation, apoptosis, and cell cycle of glioma cells by cell biological function assays. All in all, we performed bioinformatics analysis to determine the prognostic value of CDK5 on gliomas. We found that CDK5 was associated with tumor metabolism and CDK5 knockdown inhibited cell proliferation and colony forming ability, and promoted apoptosis and cell cycle arrest. Our study could make contributions to better understanding of the mechanism of glioma development and progression.

Our analysis has some limitations. First, the number of normal brain tissues used as controls in TCGA was small, therefore, additional studies with balanced sample sizes must be performed. Second, we adopted only one glioblastoma cell line for the cell biological function assays. Finally, GSEA showed that several metabolism-related pathways were enriched in the high CDK5 expression phenotype group. To validate this and fill the knowledge gap in the oncogenic mechanism of CDK5 in glioblastoma, more cell and animal experiments must be performed.

## Conclusions

Our study revealed that CDK5 expression levels were higher in glioblastoma than in LGG. We also found that CDK5 was related to adverse outcomes and tumor metabolism. Taken together, CDK5 might act as a potential prognostic biomarker and a tumor metabolism target for glioma. We further explored the role of CDK5 in human U251MG glioma cells via CDK5 knockdown. CDK5 knockdown impaired cell proliferation and colony formation ability and induced apoptosis and cell cycle arrest. Overall, CDK5 might prove to be a valid target for glioma therapy.

## Supplementary Material

Supplementary table S1.

## Figures and Tables

**Table 1:** Association between CDK5 expression and the clinicopathological characteristics (logistic regression) of gliomas in TCGA and GSE16011

Clinical characteristics	TCGA			GSE16011		
	Total (N)	Odds ratio (95%CI)	P-value	Total (N)	Odds ratio (95%CI)	P-value
Age (≥52 vs. <52)	670 (263 vs. 407)	2.47 (1.80-3.41)	<0.001	276 (133 vs. 143)	1.85 (1.15-2.99)	0.0117
Sex (male vs. female)	670 (386 vs. 284)	0.822 (0.605-1.12)	0.211	276 (184 vs. 92)	1 (0.606-1.65)	1
Vital status (dead vs. alive)	670 (220 vs. 450)	2.36 (1.69-3.29)	<0.001	264 (240 vs. 24)	2.64 (1.10-7.04)	0.0378
Grade (IV vs. I+II+III)	670 (161 vs. 509)	6.53 (4.28-10.2)	<0.001	276 (159 vs. 117)	1.88 (1.16-3.05)	0.011
**Histological type**						
(GBM vs. pilocytic astrocytoma)				166 (159 vs. 8)	64.3 (1.03e-06-NA*)	0.984
(GBM vs. astrocytoma)	352 (160 vs. 192)	9.87 (6.09-16.4)	<0.001	188 (159 vs. 29)	1.46 (1.10-1.98)	0.0111
(GBM vs. oligoastrocytoma)	288 (160 vs. 128)	4.65 (2.83-7.74)	<0.001	187 (159 vs. 28)	0.988 (0.439-2.22)	0.976
(GBM vs. oligodendroglioma)	350 (160 vs. 190)	5.85 (3.71-9.38)	<0.001	211 (159 vs. 52)	1.10 (0.804-1.51)	0.549
Tumor status (with tumor vs. tumor free)	591 (400 vs. 191)	1.72 (1.21-2.44)	0.002			
IDH1 mutation (yes vs. no)	125 (91 vs. 34)	0.428 (0.185-0.954)	0.0414	226 (83 vs. 143)	0.963 (0.560-1.65)	0.890
KPS (<80 vs. ≥80)	412 (71 vs. 341)	2.82 (1.65-4.99)	<0.001	265 (82 vs. 183)	0.922 (0.546-1.55)	0.759
Ethnicity (Hispanic or Latino vs. not Hispanic or Latino)	609 (34 vs. 575)	0.342 (0.149-0.720)	0.007			
Family history of cancer (yes vs. no)	338 (132 vs. 206)	1.35 (0.871-2.09)	0.181			

*NA, not available.

**Table 2:** Gene sets enriched in high *CDK5* expression phenotype

MSigDB collection	Gene set name	NES	NOM p-val	FDR q-val
Kegg.v6.2.symbols.gmt	KEGG_AMINO_SUGAR_AND_NUCLEOTIDE_SUGAR_METABOLISM	1.81	0.00204	0.0364
	KEGG_GALACTOSE_METABOLISM	1.89	0.00390	0.0204
	KEGG_GLUTATHIONE_METABOLISM	1.95	0	0.0154
	KEGG_GLYOXYLATE_AND_DICARBOXYLATE_METABOLISM	1.76	0.00202	0.0450
	KEGG_OXIDATIVE_PHOSPHORYLATION	2.08	0	0.00674
	KEGG_PENTOSE_PHOSPHATE_PATHWAY	1.76	0	0.0480
	KEGG_PROTEASOME	1.91	0	0.0193

NES: normalized enrichment score; NOM: nominal; FDR: false discovery rate. Gene sets with NOM P-value <0.05 and FDR q-value <0.05 were considered as significantly enriched.
