# Supplementary material for: CDK5 Knockdown inhibits proliferation and induces apoptosis and Cell Cycle Arrest in Human Glioblastoma
Source: J Cancer. 2021 May 10;12(13):3958–66. doi: 10.7150/jca.53981 (PMC8176241; doi:10.7150/jca.53981)
Supplement: Supplementary file 1 — Supplementary table S1. [file jcav12p3958s1.pdf]

Table S1. Glioma patients' characteristics in TCGA and GSE16011.

Clinical characteristics		TCGA		GSE16011	
		No. of patients	Percentage (%)	No. of patients	Percentage (%)
**Age (year)**		9-89 (Median 52)		11-81 (Median 51)	
**Sex**	Male	651	58.4	184	66.7
	Female	460	41.3	92	33.3
	Missing	3	0.270	0	0
**Vital status**	Alive	570	51.2	24	8.70
	Dead	539	48.4	240	87.0
	Missing	5	0.450	12	4.30
**WHO grade**	G1			8	2.90
	G2	249	22.4	24	8.70
	G3	265	23.8	85	30.8
	G4	596	53.5	159	57.6
	Missing	4	3.60	0	0
**Histology**	Pilocytic astrocytoma			8	2.90
	Astrocytoma	194	17.4	29	10.5
	Oligodendroglioma	191	17.2	52	18.8
	Oligoastrocytoma	130	11.7	28	10.1
	Glioblastoma	596	53.5	159	57.6
	Missing	3	0.270	0	0
**KPS**	<80	151	13.6	82	29.7
	≥80	584	52.4	183	66.3
	Missing	379	34.0	11	3.99
**Tumor status**	Tumor free	209	18.8		
	With tumor	783	70.3		
	Missing	122	10.95		
**IDH1 mutation**	Yes	91	8.17	83	30.1
	No	34	3.05	143	51.8
	Missing	989	88.8	50	18.1
**Family history of cancer**	Yes	132	11.9		
	No	210	18.85		

	Missing	772	69.30
**Ethnicity**	Hispanic or Latino	45	4.04
	Not Hispanic or Latino	939	84.29
	Missing	130	11.67
